# A Multiscale Mapping Assessment of Lake Champlain Cyanobacterial Harmful Algal Blooms

**DOI:** 10.3390/ijerph120911560

**Published:** 2015-09-15

**Authors:** Nathan Torbick, Megan Corbiere

**Affiliations:** 1 Applied Geosolutions, Newmarket, New Hampshire, NH 03857, USA; E-Mail: mcorbiere@appliedgeosolutions.com; 2 Department of Neurology, Dartmouth College, Hanover, New Hampshire, NH 03755, USA

**Keywords:** cyanobacterial harmful algal blooms, Lake Champlain, Landsat 8 OLI, RapidEye, Proba CHRIS, water quality, phycocyanin, chlorophyll-a

## Abstract

Lake Champlain has bays undergoing chronic cyanobacterial harmful algal blooms that pose a public health threat. Monitoring and assessment tools need to be developed to support risk decision making and to gain a thorough understanding of bloom scales and intensities. In this research application, Landsat 8 Operational Land Imager (OLI), Rapid Eye, and Proba Compact High Resolution Imaging Spectrometer (CHRIS) images were obtained while a corresponding field campaign collected *in situ* measurements of water quality. Models including empirical band ratio regressions were applied to map chlorophyll-a and phycocyanin concentrations; all sensors performed well with R^2^ and root-mean-square error (RMSE) ranging from 0.76 to 0.88 and 0.42 to 1.51, respectively. The outcomes showed spatial patterns across the lake with problematic bays having phycocyanin concentrations >25 µg/L. An alert status metric tuned to the current monitoring protocol was generated using modeled water quality to illustrate how the remote sensing tools can inform a public health monitoring system. Among the sensors utilized in this study, Landsat 8 OLI holds the most promise for providing exposure information across a wide area given the resolutions, systematic observation strategy and free cost.

## 1. Introduction

Concern over toxins and public health threats resulting from cyanobacterial harmful algal blooms (CHABs) have gained attention as reoccurring and seasonal blooms persist in many waters. Cyanobacteria are particularly noxious when anthropogenic eutrophication of water bodies causes large concentrations to form blooms. While blooms often receive notoriety, the health impacts of chronic exposure to low or moderate levels of cyanotoxins is not fully known. Characterization of cyanobacteria and their toxins has a long and, at times, divergent history. Broadly, cyanotoxins can be described as having negative health impacts and can be grouped by the chemical structure, tissue or target systems (e.g., neurotoxic, hepatotoxic, dermatotoxic). Cyanotoxins found within inland freshwater lakes include saxitoxins, anatoxin, cylindrospermopsins, lyngbyatoxins and microcystins, which tend to be the most frequently reported [1,2,3]. Many variants exist; not all cyanobacteria produce toxins; and different cyanobacteria can produce similar cyanotoxins. For example, *Microcystis*, *Anabaena*, *Aphanizomenon*, *Anabaenopsis*, *Nostoc* and *Planktothrix* all have been shown to be capable of producing microcystins. 

The neurotoxin β-N-methylamino-L-alanine (BMAA) can be produced by cyanobacteria and has been associated with CHABs and amyotrophic lateral sclerosis (ALS) clusters across northern New England [4,5,6,7]. An ALS cluster of higher than expected normalized incidence happens to occur by Lake Champlain adjacent to bays where reoccurring CHABs have been observed. Recent research has shown that pathogenic mechanisms in sporadic and familial ALS cases include protein misfolding [8,9,10,11], which is probably the major mechanism by which the cyanobacterial neurotoxin, BMAA, produces chronic neurotoxicity [12] and impairment of ribonucleic acid (RNA) metabolism [13]. It is possible that humans may be exposed to the cyanotoxins produced by cyanobacteria via the food chain, drinking water, aerosolization and by recreational use of waterbodies [5]. There have also been reported dog deaths attributed to cyanotoxins in Lake Champlain CHABs. As the health implications of CHABs raises concern, monitoring and assessment tools are required to support decision making. 

Lake Champlain currently has a tiered alert system for cyanobacteria monitoring based on a protocol coordinated across multiple organizations. This includes volunteers, environmental state agencies (*i.e*., Vermont Department of Environmental Conservation (VT DEC)), state health agencies (*i.e*., Vermont Department of Health (VT DH)), several key non-governmental organizations and high level international agreements being on the borders of Vermont, New York and Canada. The assessment protocol is a hierarchical framework that increases in scrutiny as reports, sampling, screening and toxin testing criteria are met. Shoreline and within lake samples are systemically collected every two weeks at designated stations for established attributes. Numerous local reports of “visual conditions” from volunteers and the public are also integrated. The tiered system is triggered if a scum, highly discolored water, foul odor or potential CHAB indicator is reported or an *in situ* sample shows indications of a CHAB. Once triggered, toxin and phytoplankton samples are obtained. Initial qualitative screening identifies the location, and 3-m vertical plankton tows (63-μm mesh) are collected for microscopic analysis by experts [14,15,16] within 72 h. Identification is carried out for assessment of potential toxin-producing cyanobacteria. However, if high algal densities were measured in the previous sample or if a surface scum is present, a surface grab will also be collected and tested for potential toxin-producing cyanobacteria.

If potential toxin-producing cyanobacteria are enumerated as being over 2000 cell/mL in the upper 3 m of the water column, another round of sampling is triggered. If the next round (“vigilance”) of potentially toxic taxa cell densities exceed 4000 cell/mL then enzyme-linked immunosorbent assays (ELISA) for toxin (microcystins) analyses are executed and an Alert Level 1 status is set in place. Anatoxins are analyzed when the colony consists largely of producers believed to generate said toxin. If ambient toxin concentrations are >6 µg/L microcystins, an Alert Level 2 status is put in place. It is feasible that anatoxins are missed due to time-consuming techniques and since ELISA does not screen for anatoxins. Dense or visible scums can also trigger an Alert Level 2 status. As conditions improve, the alert system retreats to lower levels of concern.

Satellite remote sensing has been used as an effective tool to derive information on inland lake water quality [17,18,19,20,21,22,23,24,25,26,27,28,29,30,31,32]. Sensor characteristics vary across satellites, and no single satellite platform provides all of the optimal resolutions required for comprehensive monitoring and assessment of CHABs. Tradeoffs balancing spatial resolution or pixel size, temporal overpass frequency, radiometric sensitivity and spectral wavelengths, as well as availability and cost must get considered when choosing a sensor. These choices also influence the algorithm or mapping approach that is most effective for a given application. Mapping approaches generate products from bio-optical algorithms, which can be empirical (“statistical”) or analytic (“based on spectral properties”) in form. Both approaches have strengths and limitations that are directly linked to the resolutions and given application. These two broad categories are not mutually exclusive, as some empirical models have been developed from techniques using radiative transfer equations, and most analytical algorithms contain empirical coefficients [33]. Further, fusion techniques blending the strengths of each approach may help provide a more thorough assessment. Therefore, the parameter of interest, scale, method and sensor are intertwined for lake monitoring, and no one sensor or approach is comprehensive and optimal for mapping lake CHABs.

A few studies have utilized satellite remote sensing to map water quality in Lake Champlain. Trescott [34] developed empirical regression models using selected *in situ* data with corresponding Landsat bands and ratios based on blue, green and red bands. Results for predicting Secchi depth, chlorophyll-a (chl-a) and cyanobacteria biovolume are reported with *R^2^* of 0.78, 0.81 and 0.81, respectively. Wheeler *et al.* [35] developed QuickBird band ratio algorithms, which achieved *R^2^* 0.68 for phycocyanin concentrations (PC). They reported that MEdium Resolution Imaging Spectrometer (MERIS) semi-analytical algorithms (e.g., [36,37,38]) achieved high overall accuracy while noting underestimation of phycocyanin at higher (>80 µg/L) concentrations when mapping Missisquoi Bay. Lunetta *et al*. [39] evaluated the use of MERIS and the “Cyanobacteria Index” (e.g. [29,40]) to derive cyanobacteria cell count (cells/mL) across eastern USA lakes. They found the approach worked well for low (10,000 to 109,000) and very high (>1,000,000) concentrations; however, note that for intermediate concentrations, the approach had substandard performance. Scale, temporal dynamics, conditions, attenuation and composition of phytoplankton community are common confounding themes across the studies. 

The goals of this study were to evaluate strategic sensors, to carry out multiscale mapping of CHAB in strategic bays in Lake Champlain and to illustrate how satellite remote sensing can support public health. Sensors evaluated include the Landsat 8 OLI, RapidEye and Proba-1 CHRIS considering the spectral coverage, cost effectiveness, spatial resolution and scale, as well as the availability. A coordinated campaign was executed in Malletts and St. Albans Bays of Lake Champlain with the latter undergoing reoccurring CHABs. The outcomes are geared to support public health decision making and eco-epidemiological risk modeling to understand the potential impacts of CHABs and toxins on human health. 

## 2. Methods

### 2.1. Lake Champlain

**Figure 1:**
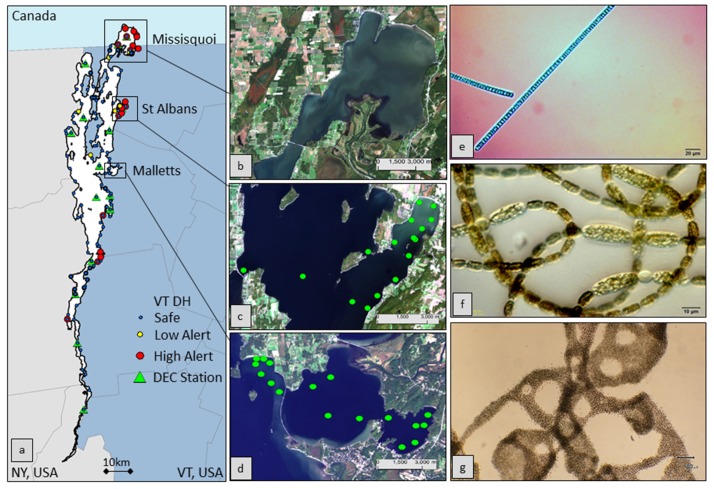
(**a**) Lake Champlain (left) location with the Vermont (VT) Department of Health (DH) 2014 sample sites and Department of Environmental Conservation (DEC) 2014 sampling stations highlighted and color coded by status. The middle panel shows noteworthy bays, (**b**) Missisquoi, (**c**) St Albans and (**d**) Mallets, with green dots showing *in situ* collection sites. The right panel shows (**e**) *Oscillatoria*, (**f**) *Anabaena* and (**g**) *Microcystis* cyanobacteria examples found in the region (microscopic images courtesy of the University of New Hampshire Center for Freshwater Biology).

Lake Champlain resides in the Eastern Great Lakes Lowland ecoregion on the borders of Canada, New York and Vermont, USA (Figure 1). Due to the geographic positioning, the lake has a complex socioecological system with several noteworthy international agreements related to trade, water quality and ecosystem management. More than 500,000 people reside in the watershed that covers more than 21,326 km^2^ of mixed forests, agriculture and urban, rural and suburban transition zones. Approximately 200,000 people depend on Lake Champlain for drinking water, with more than 100 systems drawing water for consumption. There are 54 public beaches and hundreds of private homes and recreational beaches. The lake has a large fishing and recreation community with the lake economy projected at more than $4 billion annually. The lake has a volume of 25.8 km^3^ with a surface area of 1127 km^2^ that stretches 193 km north-to-south and 19 km east-to-west with average depths of 19 m and a maximum depth of 122 m. More than 70 significant islands dot the lake, including many that host seasonal camps. Lake Champlain can stratify in warmer seasons with an epilimnion extending down to around 10 m in the main lake basin. The region has mostly a humid continental climate with warm summers and cold winters. 

### 2.2. Field Data Collection

A field campaign was designed to spatially sample a range of lake conditions considering cyanobacterial concentrations, lake depth, wind exposure and logistics. The field campaign timing and satellite overpass schedule were coordinated as best as logistically feasible. The campaign included two days of direct sampling carried out on Days of Year 251 and 252 (8 and 9 September) of 2014. Cruise sampling transects targeted St Albans and Malletts Bays to serve as a paired problematic bay undergoing chronic CHABs and a comparative and relatively pristine reference bay, respectively (Figure 1). Fifteen samples focused on St Albans Bay, and seventeen focused on Malletts Bay. Sampling also included points in Great Back Bay, the near shore adjacent to the Sand Bar National Waterfowl Management Area and Knight Point State Park, the Narrows and Eagle Bay. 

A multi-parameter sonde from YSI was used to measure chlorophyll-a, cyanobacteria concentrations, dissolved oxygen, fluorescent dissolved organic matter (FDOM) as a surrogate for chromophoric (colored) dissolved organic matter (CDOM) and a suite of other parameters (e.g., temperature, total dissolved solids, conductivity). The “cyanobacteria” sensor measures phycocyanin pigments using *in vivo* fluorometry (IVF) in real time detecting biomass concentrations with a resolution of 1 cell/mL (0.1 Relative Fluorescence Units). The total algae sensor contains excitation beams at 470 nm (blue) that resolve chlorophyll-a molecules and excite phycocyanin pigment. The probes were calibrated using standards and laboratory samples that were extracted to ensure accuracy and cross-validation of plankton tows. The sonde was ported to a handheld integration device to simultaneously record Global Positioning System values and instrument observations. Strategic depth profiles were also obtained to understand stratification and water column properties as related to the remote sensing components and lake conditions. 

**Table 1:** Sensor characteristics utilized in this research application.

Sensor	Spatial Resolution	Bands	Spectral	Footprint	Overpass Day Of Year
Landsat 8 OLI	30	11	VNIR, MIR, Thermal	185 × 185 km	267
Proba-1 CHRIS	18	19	VNIR	13 × 13 km	265
RapidEye	5	5	VNIR	25 × 25 km	260

### 2.3. Landsat 8 OLI

**Figure 2:**
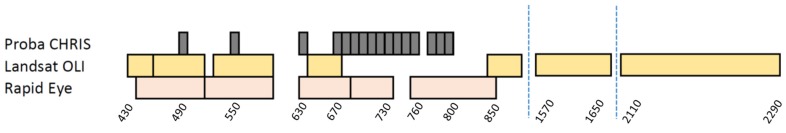
Spectral bands and wavelength (nm) regions observed from the three sensors with dotted blue hashes indicating breaks in the scale.

Landsat 8 Operational Land Imager (OLI) was launched into orbit 11 February 2013 as the next generation of the Landsat Data Continuity Mission (LDCM). OLI collects seven spectral bands at 30-m spatial resolution between 430 and 2290 nm, one panchromatic (500 to 680 nm) band at 15 m and two thermal infrared (TIRS) channels (10,600 to 11,190; 11,500 to 12,510 nm) at 100-m resolution (Table 1, Figure 2). Landsat 8 has improved signal-to-noise radiometric performance with spectral coverage quantized over a 12-bit dynamic range. To the best of our knowledge, this study represents one of the first times Landsat 8 OLI has been tested or applied for CHABs assessment. 

Level 1T terrain-corrected imagery for path row 014029 on Day of Year (DOY) 267 (24 September 2014) were obtained from U.S. Geological Survey Earth Explorer. Standard processing was performed at USGS, including resampling using cubic convolution and projection into Universal Transverse Mercator zone 18-north. We evaluated preprocessing and transformations following the lineage of Torbick *et al.* [31]. This included evaluating the use of water leaving radiance using gain and offsets (Rad), top-of-atmosphere (TOA) reflectance normalized by the exoatmospheric solar irradiance and Sun angle and surface reflectance (SR) using 6S parameterized with Moderate Resolution Imaging Spectroradiometer (MODIS) Aerosol Optical Depth (AOD), similar to the Landsat Ecosystem Disturbance Adaptive Processing System (LEDAPS) approach (e.g., [41,42]). The model selected for final mapping of CHAB concentration centered on the use of atmospherically-corrected surface reflectance for potential transferability and multitemporal mapping. For storage and processing purposes, a gain of 0.0001 was applied for Landsat. The scene had no clouds or shadows near the lake; therefore, further cloud processing was not required. 

### 2.4. RapidEye

RapidEye is commercial satellite imaging company that provides relatively cost-effective multispectral fine-scale (5 m) imagery in the visible to near-infrared (VNIR) domain. Since the red edge is often emphasized for inland waters, the utility of the platform was considered for this application. The RapidEye constellation is made up of five platforms capable of observing locations with five day revisits at nadir and daily revisits if off nadir with multispectral push broom sensors that orbit at 630 km in a Sun-synchronous pattern. Processed images have an orthorectified pixel spacing of 5-m ground resolution. Data are 16-bit unsigned integers covering the blue (440 to 510 nm), green (520 to 590 nm), red (630 to 685 nm), red edge (690 to 730 nm) and NIR (760 to 850 nm) portions of the spectrum. 

Images were delivered as orthorectified tiles with radiometric and geometric corrections applied to the Level 1B data. No further geometric corrections were required. Radiance, TOA reflectance and surface reflectance using 6S parameterized with MODIS AOD were computed. The final RapidEye model selected for mapping of CHAB concentration also focused on the use of atmospherically-corrected surface reflectance for potential transferability and multitemporal mapping. St Albans and Malletts Bays were collected on the same DOY (260) and, thus, required no additional normalization for model building. However, seven scenes north-to-south and two scenes east-to-west are required for gapless and wall-to-wall coverage of Lake Champlain. In order for a cloud-free wall-to-wall mosaic, a total of 16 scenes that included 25 August 2014, 15 September 2014 and 17 September 2014 were collected. Using the target bays and optimal conditions, we applied a dark object subtraction approach to adjust imagery taken on other dates. Overlapping regions of clear water were identified in neighboring imagery, and values were subtracted in a “wall papering” approach that used feathering. 

### 2.5. PROBA-1 CHRIS

The Compact High Resolution Imaging Spectrometer (CHRIS) flies aboard the Project for On-Board Autonomy (Proba). Proba launched in 2001 into a Sun-synchronous elliptical polar orbit at 600 km. CHRIS was intended to serve as a low cost prototype for high spectral and spatial resolution platforms beginning to bridge the gap between airborne and spaceborne imaging. The platform is unique in its compact payload, across-track pointable capability in that a target can be imaged with multiple viewing angles (−55°, −36°, 0°, 36° and 55°) within minutes and programmability. We collected Mode 4, which provides 18 spectral bands from 486 to 805 nm at a spatial resolution of 17-m pixels within a 14-km footprint over our target area. Mode 4 is similar to Mode 2; however, it does not have a 411- or 442-nm channel and does not extend as far on the NIR edge, but does have several more narrow bands in the red and NIR edge area (10 channels across 703 to 792 nm), which we sought to consider for this application and lake conditions. Two scenes over the target bays were obtained on DOY 265.

We used the open source BEAM VISAT software Version 4.10.3 and the CHRIS toolbox designed to preprocess the Proba CHRIS imagery [43,44] following the lineage described in Casal *et al.* [45]. Drop out due to the entrance slit of the spectroradiometer and vertical striping typical of push broom sensors were first corrected using the Gomez-Chova *et al.* [46] algorithm (e.g. [47,48,49]). Next, atmospheric correction was performed using look-up tables based on moderate resolution transmittance (MODTRAN) with dependencies for view zenith angle, solar zenith angle, relative azimuth angle, surface elevation and aerosol optical thickness at 550 nm. More details on the atmospheric correction procedures can be found in the Algorithm Theoretical Basis Document (ATBD) designed for the CHRIS module in BEAM [50]. 

### 2.6. Analytical Approach

*In situ* data points were buffered to create a 3 × 3 pixel array corresponding to the spatial scale (pixel size) of the respective sensor (e.g., 90-m buffer for 30-m Landsat 3 × 3 pixel array). The average value for the 3 × 3 array was extracted from all bands for each of the three sensors. Values were compiled for each sensor and linked to the *in situ* lake sampling outcomes. Strategic models were then built in the R statistical software. This work focused on utilizing empirical models considering the broad multispectral bands of Landsat and RapidEye. Bands, band ratios and band combinations shown to have spectral relationships with water quality properties in previous studies were considered as independent variables. Additionally, a subsample of known estimation algorithms and shape filter derivative indices that are transferable to the Proba CHRIS spectral configuration were also considered to evaluate the utility for this application (e.g., the Cyanobacteria Index [39], normalized indices [51,52], MER-3B and NASA’s OC4, all tuned to the nearest CHRIS spectral bands, respectively). 

The evaluation of different algorithms was carried out following methods described in Torbick *et al*. [31]. In summary, bands and ratios were systematically added, paired and removed while examining statistical performance, residuals and information theoretic criteria (*i.e*., adjusted *R^2^*, significance values, RMSE, Akaike information criterion (AIC)) for predicting chl-a and PC observations. Models with high adjusted *R^2^*, low RMSE and high relative quality, as indicated by AIC, were desired. 

Models and cross-validation were executed using withheld samples to assess robustness and prediction performance in an out-of-sample fashion following Torbick *et al*. [6] and Torbick *et al*. [31]. More complex classification and regression trees, neural net models or fusion techniques were not considered for this application, since the sample size was not extremely large (*n* = 32), the and interpretation of the inputs and corresponding results for assessing outcomes across the different sensors for risk assessment was an underlying objective. Scatterplots using out-of-sample predictions, histograms and box and whisker plots of map values in bays were each thoroughly examined. Ultimately, we considered the explanatory power of the variables, the comparison against known algorithms and the benefits of more straightforward models *vs*. models with higher accuracy, but more complexity to pick the optimal model for mapping CHABs using the three sensors.

**Figure 3:**
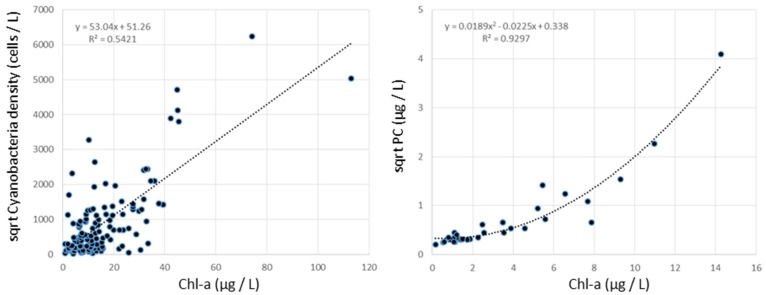
Scatterplots between transformed cyanobacteria density and chl-a collected by VT DEC (**left**) showing moderate relationship and transformed phycocyanin concentration (PC) and chl-a (**right**) collected in this application in the paired target bays showing a strong relationship.

As a final task, we generated an example alert status that complements the current hierarchical CHABs decision making framework by utilizing the derived remote sensing models and historical field data collected in Lake Champlain by the VT DEC. The VT DEC long-term monitoring program differs from the cyanobacteria monitoring program organized by the VT DH in that the VT DEC cyanobacteria density is a full cell count of all cyanobacteria within the sample, and the VT DH density is a rapid assessment of only the potentially toxic species of cyanobacteria. The VT DH will also use surface grabs occasionally, often resulting in a higher cyanobacteria density than observed by the long-term monitoring program. A subset of VT DEC samples with simultaneous chlorophyll-a (µg/L) and cyanobacteria density (cells/L) measured across the fifteen possible sampling stations in Lake Champlain at a depth of less than 3 m between 1992 and 2013 was used (*n* = 185). Models between *in situ* chl-a and enumerated cyanobacteria density were derived with a straightforward linear model having a modest correlation. Both the VT DEC chl-a *vs*. cyanobacteria density relationship and the extrapolated remote sensing models have uncertainty (Figure 3). Therefore, we emphasize the intended use of these alert status maps is to provide an indication of potential health threats and to devise ordinal classes based on the tiered alert system to show how remote sensing might support a CHABs alert decision support system.

## 3. Results and Discussion

### 3.1. Sensor Evaluation

**Figure 4:**
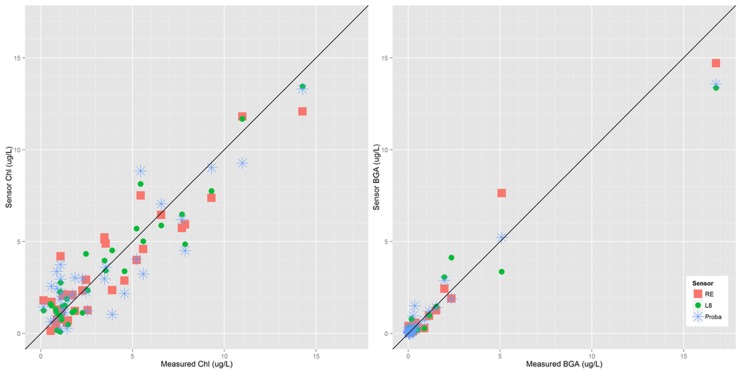
Scatterplots between observed and modeled chlorophyll-a and phycocyanin concentration (BGA) across Landsat 8 OLI (L8), RapidEye (RE) and Proba CHRIS (Proba).

All three satellite sensors showed a moderate to strong ability to model chl-a and PC observations from St Albans and Malletts Bays. Selected band ratio regression models using surface reflectance are shown in Table 2 and corresponding scatterplots in Figure 4. Landsat 8 OLI, Proba CHRIS and RapidEye had an adjusted R^2^ of 0.83, 0.88 and 0.77 for PC and of 0.77, 0.88 and 0.81 for chl-a, respectively. Landsat 8 and Proba had relatively low RMSE values (μg/L) of 0.41 and 0.54 for chl-a models compared to that of RapidEye of 1.46. RapidEye had the highest RMSE for PC models of the three sensors. Use of the different pre-processing levels had only slight differences on the outcomes, significant independent variables and did not change among processing levels. Since the potential for long-term mapping over time exists, we focused model implementation on surface reflectance considering transferability. All three sensors tended to slightly underestimate the highest PC observation in St Albans Bay, while Proba had the lowest RMSE of 1.02 followed by Landsat 8 and RapidEye with 1.33 and 1.52, respectively. 

**Table 2:** Selected empirical models highlighting the prediction results.

Metric	Sensor	Model	Adj R2	RMSE
chl-a	Landsat 8 OLI	− 59.33 + B4/B2 (34.7) + B5 (0.006)	0.77	0.41
chl-a	Proba-1 CHRIS	−4.26 + B1 (−338.6) + B2/B1 (−0.9) + B2 (682.9) + B15 (−939.1)	0.88	0.54
chl-a	RapidEye	−2.84 + B1 (−0.05) + B3 (0.08)	0.81	1.46
PC	Landsat 8 OLI	− 2.85 + B1 (0.013) + B3 (−0.43) + B4 (0.76)	0.83	1.33
PC	Proba-1 CHRIS	6.2 + B2 (334.8) + B6 (− 1644.3) + B8 (2031.6) + B11 (−709.4) + B14 (−1324.3)	0.88	1.02
PC	RapidEye	− 56.13 + B3 (0.12) + B1/B3 (9.49)	0.77	1.52

The “semi-analytical” algorithms evaluated in this application did not outperform the empirical models built for the corresponding *in situ* data. The Cyanobacteria Index, MER3-B, OC4 or other algorithms tuned to Proba CHRIS generally showed only a modest ability to predict outcomes in this case. Further, algorithms focused around 590, 620 or 708 nm had modest outcomes in this case. The newer coastal Landsat 8 OLI band did not surpass the traditional band combinations widely used in previous inland applications, including lakes in China [30], Maine [24], Michigan [31], Minnesota [53], northern New England [6], Tennessee [54] and Wisconsin [18]. However, the new, narrow Band 5 NIR (850 to 880) was used in the chl-a model and supports the notion that inland Case 2 waters (“optically complex”) are better served with red-NIR wavelengths. 

Not surprisingly, the Proba CHRIS spectral options outperformed both Landsat and RapidEye in terms of accuracy and precision. The Proba data tended to produce the most speckle-like noise out of the three sensors, while RapidEye tended to have some large shifts in adjacent pixels. This is likely related to the scale of processes and patterns in water. 

Clearly, the 5-m spatial resolution of RapidEye was superior to that of the Landsat or Proba pixel size. RapidEye was able to map into smaller channels and coastlines, whereas the other sensors were limited for finer scale details. Some of the high values immediate to the coastline in Landsat can be partially attributed to a land or bottom signal having an adverse impact on the modeled variable. In the plots, a buffer was applied to the coastline as to not include any potentially spurious mixed pixel values. Considering differences in days between observations, the spatial patterns and CHAB intensities are in agreement between the three sensors. Finer spatial patterns, such as currents, effluents and wind-formed patterns are visible in the RapidEye products. The Proba footprint size largely limits its use for research applications, as operational use is not feasible.

**Figure 5:**
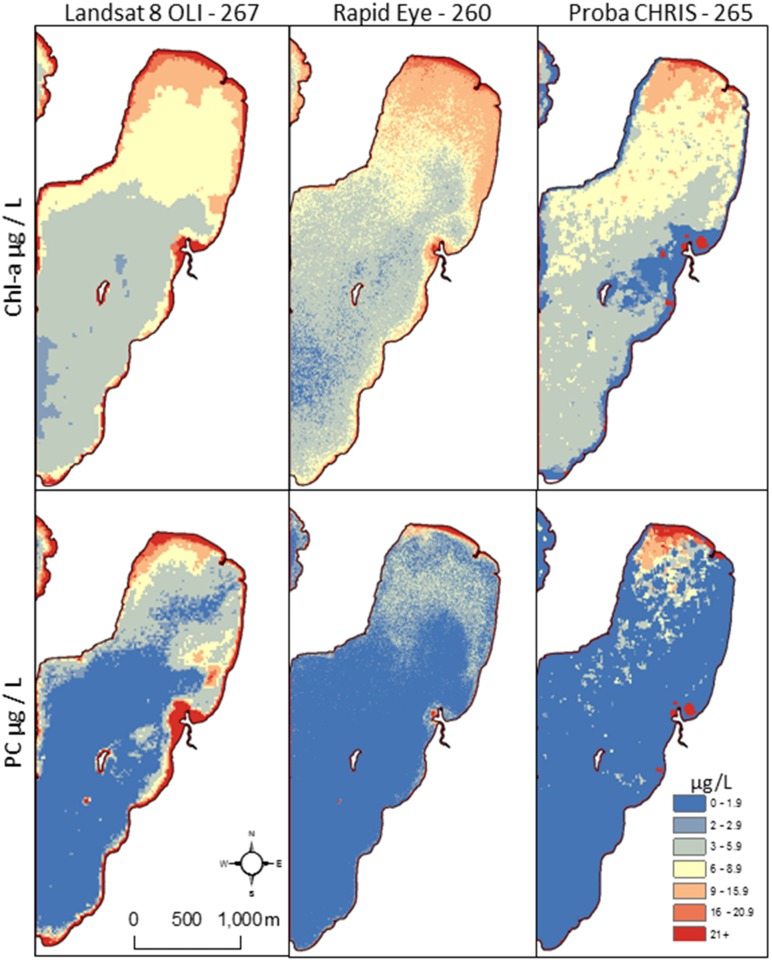
Maps of sensor predicted chl-a and PC distributions in St. Albans Bay showing generally similar spatial configurations with slightly different intensities.

**Figure 6:**
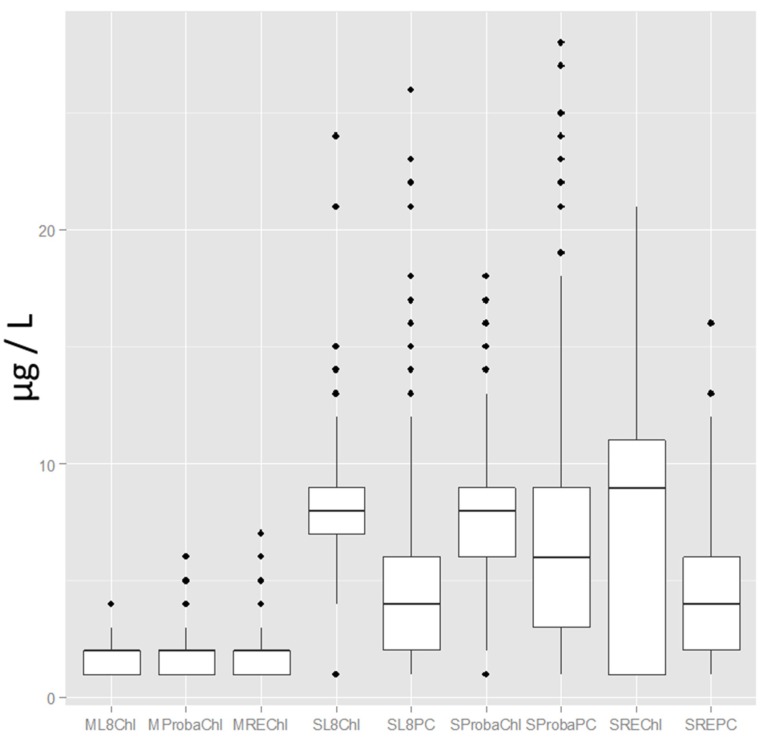
Box and whisker plots showing attributes (Chl: chlorophyll-a; PC: phycocyanin concentration) by bay (M: Malletts; S: St Albans) and satellite sensors (L8: Landsat 8 OLI; Proba: Proba CHRIS; RE: RapidEye), illustrating relatively pristine conditions in Malletts Bay and distributions of PC in St Albans Bay, as observed across the three different sensors.

RapidEye had a slightly higher mean chl-a value for St Albans Bay *versus* the other sensors (Figure 5), although a much wider distribution, as shown by the 25th and 75th percentiles in Figure 6. All three sensors showed a nearly identical distribution of low chl-a values for Malletts Bay in the box and whisker plots. Some of the wider distribution is a function of the high number of pixels given the spatial scale of the RapidEye sensors and the ability to resolve more features. The RapidEye PC outcomes are more narrowly distributed compared to Proba, which tended to have striping, even after noise reduction processing. The Proba PC distribution is a factor of the more precise and accurate model and ability to map more values. However, the processed Proba imagery was clearly sensitive to striping, and when zoomed in at a fine scale, the noise shows up in vertical patterns.

### 3.2. Alert Status Mapping

The VT DH reported eleven sampled cases in 2014 where potentially toxic cells were greater than the World Health Organization (WHO) recommended “moderate” threshold (100,000 cells/mL). This guideline is based on the potential for short-term adverse effects, including skin irritation and gastrointestinal illness. WHO guidelines suggest a tiered approach combing the use of cell counts, chl-a concentration and microcystins-LR measurements, though exact thresholds vary within the literature by date and source, which highlights the challenges and variability. These guidelines for recreational waters include low, moderate, high and very high relative probabilities for adverse health effects. No single set of guidelines currently exist in the USA, although Australia, Canada, New Zealand, Brazil, Spain, Japan and Korea, among others, note 1 ppb to 1.5 ppb microcystins (MC-LR, total microcystins) as a threshold. Several states and federal agencies in the USA are currently developing criteria likely with similar scales.

**Figure 7:**
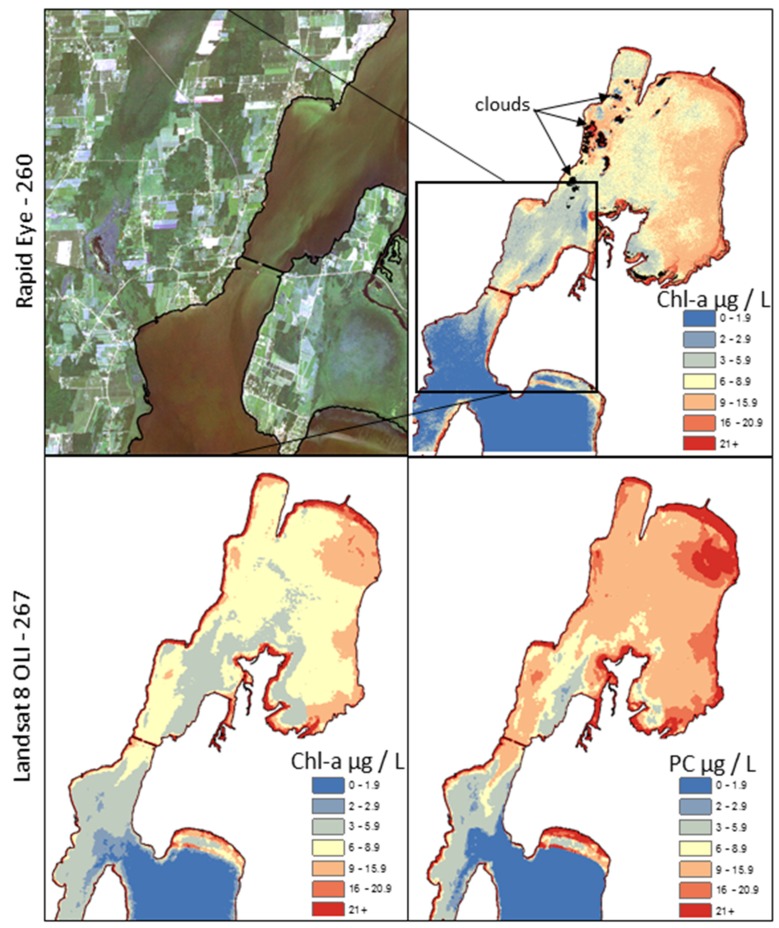
Missisquoi Bay. Top left: RapidEye image from DOY 260. Top right: chl-a density map made from the RapidEye image. Bottom left: Landsat-derived chl-a density from DOY 267. Bottom right: Landsat-derived PC density.

In Missisquoi Bay (Figure 7), the highest recording by the health department for 2014 was 2,205,200 cell/mL on 24 August 2014 (DOY 236) with microcystin and anatoxin samples reported as 2.29 and <0.5 µg/L, respectively. The most common taxa of cyanobacteria were *Anabaena, Aphanizomenon*, *Microcystis* and *Oscillatoria.* The highest potentially toxic cyanobacteria density reported by the VT DH in 2014 in St Albans Bay was 853,600 cell/mL with *Anabaena* identified as the most common taxa. Anecdotally, on the day of our field data collection, individuals were recreating in these CHAB conditions, even though the VT DH Alert Status indicated a threat. The highest concentrations of cyanobacteria density reported by VT DEC for St Albans and Missisquoi are 3,330,000 and 38,900,000 cells/L, respectively. We emphasize the differences in the methods and approach of the VT DEC and the VT DH, which make it difficult to directly compare the two datasets. 

**Figure 8:**
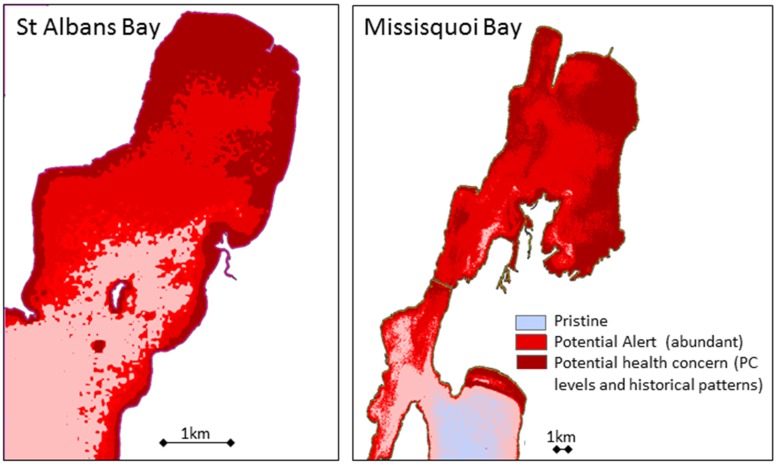
Landsat-derived alert status for two Lake Champlain bays that undergo chronic cyanobacterial harmful algal blooms (CHABs) showing potential health concerns.

The Landsat-derived alert status map illustrates conditions in St Albans and Missisquoi Bays having a modest probability of adverse health effects due to cyanobacteria concentrations at the time of overpass (Figure 8). The map was generated using a regression equation between chlorophyll-a and cyanobacteria concentration detailed in Figure 3. This example alert status map is shown using Landsat considering the operational collection strategy, free availability of Landsat and wall-to-wall coverage of Champlain from a single overpass. Protected and leeward regions tend to indicate relatively higher risk of cyanobacteria, although modest compared to past blooms. With the winds generally coming from the southwest direction, risk is highest along the coasts to the north and east, where depths also promote warmer waters. These factors are two of the reasons these bays undergo chronic CHABs. While Missisquoi Bay shows relatively high values, Maquam Bay to the south shows less risk and relatively fair conditions. This example alert status product highlights how the maps can help decision makers understand hot spots and spatial patterns that can also supplement sampling or actions related to the intensity and magnitude of CHABs. Multitemporal imagery has the potential to support frequency and duration actions, such as how long a public recreational beach should be closed for health concerns. This would of course require further development of multitemporal models.

The difference between *in situ* data collection and satellite overpass adds uncertainty and a potential error source in this application. It is possible that obtaining simultaneous *in situ* data during overpass would change the relative performance among the sensors. However, weather and lake conditions were generally similar on the three overpass days (within seven days). Coordinating overpass schedules was itself time consuming, as each sensor has a different mission. Proba CHRIS is largely an experimental research platform. A user’s request is acknowledged, and if an acquisition is planned for their task request, a notification is sent a few days before collection. This makes planning field work and coordination with other sensors very challenging. Proba images are received a few weeks to months after collection. Landsat is operational, collecting every scene (for this region) on a scheduled basis. Potential upcoming hyperspectral missions, such as the Environmental Mapping and Analysis Program (EnMAP) or the Hyperspectral Infrared Imager (HyspIRI), hold promise for providing the next generation of imaging to support water quality analysis. However, at this time, the operational use of satellites with hyperspectral instruments is limited.

Optical satellite imagery can be limited by clouds or atmosphere attenuation. The Lake Champlain region is particularly cloudy due to orographic effects and atmospheric patterns. The only other potentially usable Landsat scene (ETM+ or OLI) was collected DOY 211 (30 July), and no useful Landsat imagery for this application was taken until DOY 267. RapidEye is a commercial sensor, and users can prioritize captures if economically feasible. The ability for daily looks by RapidEye is thus superior when comparing the temporal frequencies to Landsat or Proba. High temporal frequency sensors, such as MODIS, are not capable of observing these bays due to coarser resolutions and the spatial configuration of these bays. Highlighted is the difficulty in coordinating three satellites operated by different entities, field work, lake conditions, weather and clouds. This real-world application illustrates how a program might be used by a health agency, as these data collection challenges are a reality of conducting lake CHAB remote sensing for small bays and small- to medium-sized inland lakes.

The fifty six days between usable Landsat imagery were during peak CHAB build up. Generally, in northern New England, pico- and nano-phytoplankton tend to peak during spring; green and golden phytoplankton functional groups peak late spring into the summer; and dominant potentially toxic cyanobacteria tend to bloom late summer into the fall. If cloud-free Landsat were not available for another few weeks, it is feasible that the CHAB might not have been captured. Potentially finer scale or commercial imagery can be timed to complement Landsat to inform a CHAB decision support system. 

Building direct linkages to human health decision making is a long-term vision of this application. Remote sensing tools cannot directly sense for toxins, so users must rely on surrogates (metrics) and historical patterns that represent potentially harmful conditions. More direct integration of remote sensing metrics can take place by comparing these metrics to historical conditions or known patterns along with field measurements, including toxicity and plankton tows. The “Lake Erie HAB Bulletin” produced by National Oceanic and Atmospheric Administration (NOAA) and partners is one example of how remote sensing metrics can be utilized and integrated into a larger assessment, monitoring and forecasting system. 

In the near-term, Lake Champlain and public health agencies can benefit from using regular (“operational”) remote sensing products to help inform where to sample, examine spatial variation and help understand temporal patterns. Landsat can help address this task in a cost-effective manner, though tasking multiple satellites gives the most comprehensive assessment of water quality. Figure 8 illustrated how Landsat metrics might be used to derive condition categories amenable to current health reports. Comprehensive programs will require cyanobacteria experts, citizen scientists, public health officials and environmental professionals to understand how to use the available tools to their advantage to support public health and exposure risk assessment. 

## 4. Conclusions

Landsat 8 OLI, Proba CHRIS and RapidEye all showed a moderate to strong ability to map chlorophyll-a and phycocyanin concentrations using empirical band ratio regression models. St Albans and Missisquoi Bays within Lake Champlain were mapped as undergoing CHAB conditions that may present a risk to human health. Each sensor has particular strengths and limitations related to their spatial and spectral resolutions. Landsat was chosen to illustrate an alert status map given the operational collection strategy, free availability of Landsat and wall-to-wall coverage of Champlain from a single overpass, which makes utilization by organizations more likely. The relatively large footprints, moderate spatial resolution and multispectral bands capable of resolving water quality attributes put the use of this platform to drive an operational tool within reach. Near-term Landsat metrics can be used to illustrate spatial variation, temporal cycles and relative abundance levels that can be combined with historical patterns to more thoughtfully support public health decision making. Standardization by science and end user communities, as well as more testing of transferability will be required to fully implement mapping tools for public health decision making.
